# Thyroid function and risk of sepsis: a population-based prospective cohort study with traditional and genetic epidemiological analyses

**DOI:** 10.3389/fendo.2026.1852413

**Published:** 2026-06-29

**Authors:** Marianne S. Thorkildsen, Lise T. Gustad, Bjørn O. Åsvold, Randi M. Mohus, Christina Ellervik, Alexander Teumer, Eirini Marouli, Marco Medici, Jan K. Damås, Helene M. Flatby, Tormod Rogne

**Affiliations:** 1 Department of Public Health and Nursing, Norwegian University of Science and Technology (NTNU), Trondheim, Norway; 2 Faculty of Nursing and Health Sciences, Nord University, Levanger, Norway; 3 Department of Medicine and Rehabilitation, Levanger Hospital, Nord-Trøndelag Hospital Trust, Levanger, Norway; 4 The Trøndelag Health Study (HUNT) Center for Molecular and Clinical Epidemiology, Department of Public Health and Nursing, Norwegian University of Science and Technology (NTNU), Trondheim, Norway; 5 Department of Endocrinology, Clinic of Medicine, St. Olavs Hospital, Trondheim University Hospital, Trondheim, Norway; 6 Clinic of Anesthesia and Intensive Care, St. Olavs Hospital, Trondheim, Norway; 7 Department of Clinical Biochemistry, Zealand University Hospital, Koege, Denmark; 8 Department of Laboratory Medicine, Boston Children’s Hospital, Harvard Medical School, Boston, MA, United States; 9 Department of Clinical Medicine, Faculty of Health and Medical Sciences, University of Copenhagen, Copenhagen, Denmark; 10 Department of Psychiatry and Psychotherapy, University Medicine Greifswald, Greifswald, Germany; 11 German Centre for Cardiovascular Research (DZHK), Partner Site Greifswald, Greifswald, Germany; 12 William Harvey Research Institute, Barts and the London School of Medicine and Dentistry, Queen Mary University of London, London, United Kingdom; 13 Department of Epidemiology, Erasmus Medical Center, Rotterdam, Netherlands; 14 Department of Internal Medicine, Academic Center for Thyroid Diseases, Rotterdam, Netherlands; 15 Department of Clinical and Molecular Medicine, Norwegian University of Science and Technology (NTNU), Trondheim, Norway; 16 Department of Infectious Diseases, Clinic of Medicine, St. Olavs Hospital, Trondheim, Norway; 17 Department of Community Medicine and Global Health, University of Oslo, Oslo, Norway; 18 Department of Chronic Disease Epidemiology, Yale University School of Public Health, New Haven, CT, United States

**Keywords:** infection, Mendelian randomization, population-based study, sepsis, thyroid function

## Abstract

**Introduction:**

A recent two-sample Mendelian randomization study suggested a possible causal association between hypothyroidism and selected sepsis types. We address this knowledge gap by examining the association between thyroid-stimulating hormone (TSH) levels and risk of sepsis and severe infectious diseases, using triangulation between traditional observational and Mendelian randomization (MR) analyses.

**Methods:**

Baseline characteristics and TSH-measurements were collected from adults (>20 years) at the time of participation in the prospective, population-based Trøndelag Health Study (The HUNT Study), and linked to hospital records for ascertainment of infectious diseases. Time-to-event analyses with Cox regression was used to assess the association between TSH levels and risk of sepsis, adjusting for confounders. Next, we extracted uncorrelated (R2 < 0.01) single-nucleotide polymorphisms strongly associated (p-value < 5e-8) with TSH levels from genome-wide association studies of European ancestry participants in the ThyroidOmics Consortium. Genetic associations with risk of sepsis were extracted from European ancestry participants in the UK Biobank. Secondary genetic analyses examined other measures of thyroid function (FT4, FT3, autoimmune thyroid disease and deiodinase activity) with sepsis risk, along with genetic and observational analyses of risk of lower respiratory tract infections (LRTI) and upper urinary tract infections (UUTI).

**Results:**

In the observational analyses of 45,364 subjects in HUNT there was no association between baseline normal-range TSH and sepsis risk [HR 0.98 (95% CI 0.93–1.04) per mU/L unit increase]. TSH levels <0.5 mU/L was associated with higher sepsis risk (HR 1.50, 95% CI 1.19–1.90). In the MR analyses (271,040 subjects with TSH-measurements, and 10,154 cases with sepsis), there was no association between normal-range TSH and sepsis risk [OR 1.04 (95% CI 0.98–1.10), per SD increase]. Secondary analyses supported no link between thyroid function and risk of sepsis, LRTI or UUTI.

**Conclusion:**

Variation in baseline thyroid function in the general adult population does not causally influence the risk of sepsis, LRTI or UUTI. In contrast to earlier MR work based on genetic liability to overt hypothyroidism in clinical populations, our findings indicate that mild deviations in thyroid function within and around the reference range are unlikely to be useful targets for sepsis prevention or risk stratification.

## Introduction

Thyroid dysfunction affects approximately 5% of the global population and represents a public health concern (1). Beyond metabolic roles, thyroid hormones modulate immune system cells such as T cells, B cells, and macrophages, as well as modulating cytokine production and inflammatory signaling pathways (2, 3). Elevated thyroid hormone levels may enhance the activation and proliferation of immune cells, while thyroid hormone deficiency impair immune function (4). This suggests that variations in thyroid hormone levels may influence the risk of infection.

Previous studies indicate thyroid hormone levels fluctuate during hospitalization or intensive care unit admission due to infectious diseases (5), typically with reduced levels of free triiodothyronine (T3) and possibly in thyroxine (T4) and thyroid stimulating hormone (TSH) (6). These changes are considered potential prognostic markers but likely reflect acute response to critical illness rather than prior thyroid variation (7).

Despite current evidence showing that thyroid hormone levels change during severe infectious disease, it remains unclear if they influence infection risk. Few studies have explored thyroid function and infection risk at a population level during a non-acute phase. One population-based study found that hypothyroidism was associated with increased pneumonia risk (8). This is relevant to the hypothesis about thyroid function and risk of sepsis as lower respiratory tract infection (LRTI) and upper urinary tract infections (UUTI) are common precursors to sepsis. Supporting this, a mendelian randomization (MR) study suggested that genetic liability to hypothyroidism might be a risk factor for streptococcal and puerperal sepsis (9). However, that study relied on binary thyroid diagnoses, register-defined sepsis subtypes and did not assess continuous thyroid function, all sepsis subtypes or other more common serious infections such as LRTI and UUTI in the general population.

In a previous observational study we observed no robust relationship between TSH levels measured at baseline upon participation in the HUNT2 study and risk of future hospitalization with bloodstream infection (BSI) (10). There is still a lack of studies that combine prospective population-based data with MR to clarify whether thyroid function in the general population causally influence the risk of sepsis or its common infection precursors. The MR approach may improve causal inference by minimizing confounding and reverse causation (11), leveraging the random segregation of genetic variants as a natural experiment. The triangulation method, which involves testing the hypothesis across different datasets and methods is also lacking, to test credibility and validity compared to using one method alone.

To address these knowledge gaps, we triangulated observational and genetic epidemiological analyses. First, we examined the association between baseline TSH levels and sepsis risk using population-based observational data from the Trøndelag Health Study (HUNT Study). Next, we used data from the most recent genome-wide association study (GWAS) on thyroid function (12) to analyze whether genetically predicted TSH levels were associated with the risk of sepsis. We also performed secondary analysis of UUTI and LRTI as outcomes, given their unexplored relevance as important and common precursors to sepsis.

## Methods

We followed the Strengthening the Reporting of Observational Studies in Epidemiology guidelines for MR (13) and observational studies (14). Ethical approval was granted by The Regional Committee for Medical Research for the HUNT data (ref. 2015/1204). All participants signed informed consent for participation and the use of HUNT data in research. Genetic data were retrieved from studies that had sought informed consent from their study participants, and we only used deidentified, publicly available, summary-level data, which did not require ethical review board approval.

### Data material

#### Observational data

We used data from The Trøndelag Health Study (HUNT), a large population-based health study in Norway. All individuals ≥20 years in the Nord-Trøndelag region were invited to participate in four surveys (HUNT1 to HUNT4). We used data from HUNT2 (1995–1997) and HUNT3 (2006–2008) where the participation rates were 69% and 54%, respectively. Participants self-reported on health-related questionnaires and underwent standardized clinical examination with blood sampling for instant analysis and storage. The HUNT study has been described in more detail previously (15).

Our main exposure representing thyroid function was TSH. In HUNT2, TSH measurements were collected from all women ≥40 years, a 50% random sample of men ≥40 years, and a 5% random sample of participants <40 years (10). In HUNT3, all participants had their TSH levels measured. FT4 and FT3 levels in HUNT2 and HUNT3 were only measured for small subgroups with deviant TSH levels and the use of these was not suitable for the scope of our study (16). Participants additionally self-reported thyroid disease and use of thyroid medication (levothyroxine or thionamides) in both HUNT surveys.

Serum TSH was analyzed in the Hormone Laboratory in Aker University Hospital, Oslo, for HUNT2 and at Levanger Hospital, Nord-Trøndelag Hospital Trust for HUNT3. In HUNT2 TSH was analyzed using a non-competitive immunofluorometric assay (DELFIA hTSH Ultra; sensitivity 0.03 mU/l and total analytical variation <5%) from Wallac Oy (Turku, Finland), while HUNT3 used chemiluminescent microparticle immunoassays on an Architect ci8200 from Abbott, with reagents from Architect iSystem (Abbott Ireland, Longford, Ireland; and Abbott Laboratories).Validation analyses showed no systematic differences in TSH concentrations below 5 mU/l; above this, HUNT3 values averaged 3.7% lower than HUNT2 (16).

We analyzed TSH both as a continuous exposure restricted to TSH values within 0.5–4.5 mU/L, and stratified into six categories corresponding to our prior work; <0.5 mU/L, 0.5-1.4 mU/L, 1.5-2.4 mU/L, 2.5-3.4 mU/L, 3.5-4.5 mU/L, >4.5 mU/L, using 0.5-1.4 mU/L as our reference (10). For the TSH value of 5 mIU/L, a 3.7% difference corresponds to only 0.19 mIU/L and did not affect categorization.

Sepsis was defined as explicit or implicit sepsis based on International Classification of Diseases (ICD) hospital discharge codes (**Supplementary Table 1**) (17, 18). Explicit sepsis refers to diagnosis codes directly mentioning sepsis, while implicit sepsis refers to concurrent codes for acute infection and organ dysfunction (17). UUTI and LRTI were identified by primary or secondary ICD codes, as previously described by Flatby et al. (19, 20).

Covariates included self-reported marital status (never married, married/partnered, separated/widowed/divorced); education level (low, medium, high); smoking status (never, former or current). Body mass index (BMI) was calculated as weight (kg) divided by the squared value of height (m2), using measurements collected by trained nurses during the clinical examination of HUNT2 and HUNT3 (21). Participants were categorized as having comorbid conditions if having any of the listed conditions: (i) chronic kidney disease (estimated glomerular filtration rate <60ml/min/1.73m2 in HUNT2 or self-reported chronic kidney disease in HUNT3); (ii) cardiovascular disease (prior history of acute myocardial infarction or stroke); (iii) lung disease (productive cough for more than three months yearly the past two years in HUNT2 or self-reported chronic lung disease in HUNT3); (iv) cancer (self-reported) and; (v) diabetes (self-reported).

In the main analysis we excluded participants with sepsis before the start of follow-up, those not selected for TSH measurements, those missing thyroid disease information, those with known thyroid disease at baseline and those with missing data on covariates.

#### Genetic data

MR uses genetic variants as instrumental variables to explore potential unconfounded relationships between exposures and outcomes. It leverages the random allocation of genetic variants at conception to reduce the effect of confounding and reverse causality that often disrupts the estimates in traditional observational studies. In two-sample MR, each genetic variant’s causal estimates are calculated as the ratio of its association with the outcome to its association with the exposure (Wald ratio) (22). Subsequently, these ratio estimates are combined by methods taking pleiotropy and statistical power into account. A valid genetic instrument must meet three main assumptions: a) association with the exposure; b) not associated with the outcome through confounding pathways, and c) affect the outcome only through the exposure (23).

As in the observational analyses, TSH levels were our main exposure. We extracted uncorrelated (R^2^<0.01) single-nucleotide polymorphisms (SNPs) strongly associated (p-value < 5e-8) with TSH levels from GWAS studies of European ancestry from the latest GWAS by the ThyroidOmics Consortium evaluating TSH levels within the normal range from 271,040 individuals. Effect estimates were calculated per standard deviation of TSH units (12).

From the same GWAS, using the same cut off limits we extracted genetic instruments describing other aspects of thyroid function; FT4, FT3 and TSH levels above and below reference range (12). We also considered autoimmune thyroid disease (AITD) (24) and the two subgroups SNPs for FT4 [SNPs associated with deiodenase activity (DIO) and not associated with deiodinase activity (non-DIO)] (25).

Genetic susceptibility to sepsis was collected from a GWAS of the UK Biobank (17), while a GWAS from FinnGen was added for sensitivity analysis (26). ICD codes used to identify cases in the respected cohorts are listed in **Supplementary Table 1**. Secondary analyses included SNPs associated with UUTI (20) and LRTI (19). Information on GWAS data used is summarized in **Table 1**.

**Table 1:** Overview of GWAS studies utilized in this study.

Study	Trait	Phenotype definition	Participants	SNPs (explained variance)	Cohort
Exposure					
Sterenborg et al., 2024 (12)	TSH (primary exposure)	Continuous variable of TSH levels within cohort-specific reference range (primarily the upper and lower 2.5% percentile)	Total: 271,040	242 (4.5%)	Meta analysis of 46 cohorts (HUNT, GODARTS, EPIC-Norfolk, Fenland, MGI, Regeneron)
	FT4	Continuous variable of FT4 levels within cohort specific reference range (primarily the upper and lower 2,5% percentile).	Total: 119,120	82 (1.9%)	Meta analysis of 46 cohorts (EPIC-Norfolk, Fenland, LifeLines-2)
	FT3	Continuous variable of FT3 levels within cohort specific reference range (primarily the upper and lower 2,5% percentile).	Total: 59,061	8 (0.3%)	Meta analysis of 46 cohorts (Fenland, Lifelines-2, Regeneron)
	Low TSH	Cases: TSH below cohort specific lower limit. Controls: Not defined as case	Cases: 4,469 Controls: 137,080	31 (2.7%)	Meta analysis of 20 cohorts (HUNT, ARIC, Sardinia)
	High TSH	Cases: TSH above cohort specific upper limit Controls: Not defined as case	Cases: 6,825 Controls: 146.416	8 (0.4%)	Meta analysis of 27 cohorts (HUNT, EPIC-Norfolk, WGHS)
Saevarsdottir et al., 2020 (24)	AITD	Cases: Having Graves’ disease, Hashimoto’s or other non-autoimmune hypothyroidism Controls: Not defined as case	Cases: 30,234 Controls: 755,172	31 (0.9%)	deCODE, UKB
Outcome					
Ponsford et al, 2020 (34)	Sepsis (primary outcome)	Case: Explicit sepsis diagnosis code Control: Not classified as case	Cases: 10,154 Controls: 452,764	–	UKB
Kurki et al, 2023 (26)	Sepsis	Cases: FinnGen code AB1_OTHER_SEPSIS Controls: Not classified as cases.	Cases: 10,666 Controls: 303,314	–	FinnGen
Flatby et al, 2022 (19)	LRTI	Cases: ICD9/10 codes of at least either primary or secondary diagnosis of LRTI from hospital records. Controls: Not classified as cases.	Cases: 25,320 Controls: 575,294	–	UKB, HUNT, FinnGen
Flatby et al, 2024 (20)	UUTI	Cases: ICD9/10 codes of at least either primary or secondary diagnosis of UUTI from hospital records. Controls: Not classified as cases.	Cases: 3,873 Controls: 512,608	–	UKB, HUNT, MGI

AITD, Autoimmune thyroid disease; ARIC, Atherosclerosis Risk in Communities Study; FT3, Free triiodothyronine; FT4, Free thyroixine; GODARTS, Genetic and Observational Study of the Adult Respiratory Tract Study; GWAS, Genome-wide association study; HUNT, The Trøndelag Health Study; LRTI, Lower respiratory tract infection; MGI, Michigan Genomics Initiative; RS, Rotterdam Study; SD, Standard Deviation; TSH, Thyroid stimulating hormone; UKB, UK Biobank; UUTI, Upper Urinary Tract Disease.

AITD, Autoimmune thyroid disease; ARIC, Atherosclerosis Risk in Communities Study; FT3, Free triiodothyronine; FT4, Free thyroixine; GODARTS, Genetic and Observational Study of the Adult Respiratory Tract Study; GWAS, Genome-wide association study; HUNT, The Trøndelag Health Study; LRTI, Lower respiratory tract infection; MGI, Michigan Genomics Initiative; RS, Rotterdam Study; SD, Standard Deviation; TSH, Thyroid stimulating hormone; UKB, UK Biobank; UUTI, Upper Urinary Tract Disease.

### Statistical analyses

Our main analyses included Cox regression to assess associations between non-acute baseline TSH and risk of sepsis and two-sample MR estimating the associations between genetically predicted TSH levels on sepsis risk. Secondary analyses included alternative exposures of thyroid function (FT4, FT3, AITD and deiodinase activity) and alternative infectious disease (LRTI and UUTI).

#### Observational analyses

When estimating the risk of sepsis with baseline TSH levels in our main analysis, age was set as timescale and adjusted for the following covariates: Model 1, sex; Model 2, sex, marital status, education, smoking status and BMI. Model 2 was considered the main model. Sensitivity analysis examined the risk of sepsis with TSH as a continuous variable within reference range (0.5–4.5 mU/L) and additionally adjusted for comorbid conditions (cardiovascular disease, chronic kidney disease, lung disease, cancer or diabetes) to mediate the association between our exposure and outcome.

We performed two sets of secondary analyses for the observational data. First, we investigated whether baseline TSH levels was associated with LRTI and UUTI (27). Second, we used self-reported thyroid disease (collectively and split between hypo- and hyperthyroidism) as alternative exposures describing thyroid function.

#### MR analyses

The main MR analysis was performed in R studio version 4.3.1 using the MendelianRandomization and TwoSampleMR package. Each estimate was weighted using the inverse variance approach (IVW). Our main exposure of interest was TSH levels within the normal range, and the main outcome sepsis.

Sensitivity analyses to test the robustness of our MR findings included MR-Egger (robust to pleiotropy), weighted median (robust to outliers) and weighted mode approaches (effective when at least 50% of instruments are valid). We also repeated MR using FinnGen GWAS data as an alternate outcome (26).

Secondary MR analyses evaluated risk of developing UUTI (20) and LRTI (19). We also analyzed whether genetically predicted FT4, subgroups of FT4 (DIO and non-DIO), FT3, TSH levels outside the reference range as a proxy for hypo- and hyperthyroidism, and AITD influenced sepsis risk.

## Results

### Observational analyses

**Figure 1:**
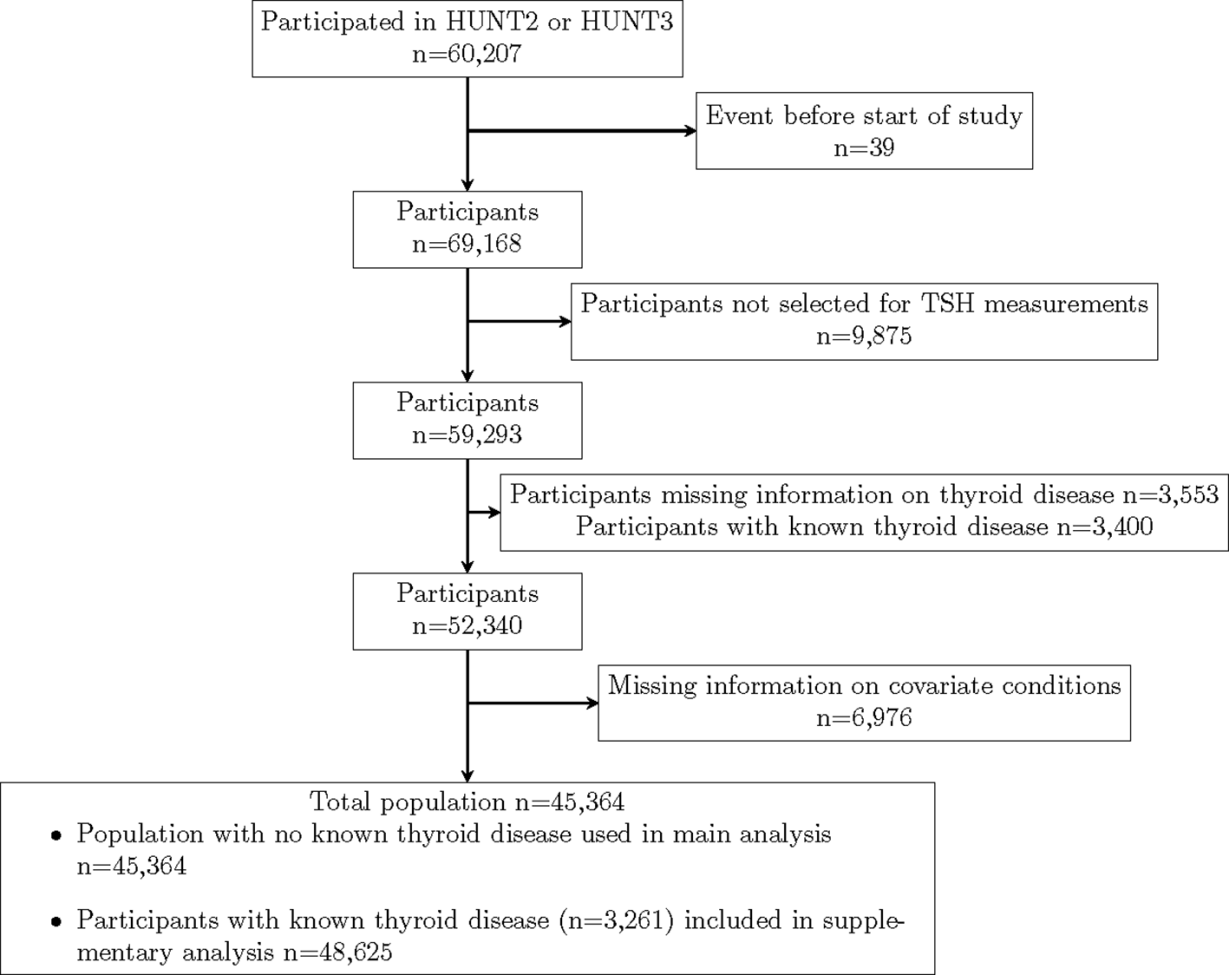
Flowchart describing the observational study.

Of the 59,293 participants with information on baseline thyroid function in HUNT2 and HUNT3, 45,364 were available for analysis (**Figure 1**). A small majority of the participants were female. Participants with TSH>4.5mU/L were older compared to the reference group 0.5–1.4mU/L, participants in the upper and lower reference range reported the highest number of comorbid conditions and participants in the TSH<0.5mUL category reported more frequently being current smokers (**Table 2**). The median follow-up time was 21 years, during which 1,927 sepsis events occurred.

**Table 2:** Baseline characteristics of the HUNT population.

TSH levels							
Variables	Total No. of subjects n (%)	<0.5 mU/L n (%)	0.5-1.4 mU/L n (%)	1.5-2.4 mU/L n (%)	2.5-3.4 mU/L n (%)	3.5-4.5 mU/L n (%)	>4.5 mU/L n (%)
Total	45,364 (100)	1,264 (2.8)	23,110 (50.9)	15,042 (33.2)	3,886 (8.6)	1,172 (2.6)	890 (2.0)
Participated in HUNT2	25,737 (56.7)	747 (59.0)	12,384 (53.6)	8,584 (57.1)	2,539 (65.3)	812 (69.3)	671 (75.4)
Participated in HUNT3	36,141 (79.7)	1,058 (83.7)	18,594 (80.5)	12,228 (81.3)	2,997 (77.1)	788 (67.2)	476 (53.5)
Participated in both HUNT2 & 3	16,514 (36.4)	415 (32.8)	8,243 (35.7)	5,612 (37.3)	1,512 (38.9)	453 (38.7)	279 (31.3)
Sex							
Female	24,846 (54.8)	857 (67.8)	12,654 (54.8)	7,876 (52.4)	2,213 (56.9)	691 (59.0)	555 (62.4)
Male	20,518 (45.2)	407 (32.2)	10,456 (45.2)	7,166 (47.6)	1,673 (43.0)	481 (41.0)	335 (37.6)
Smoking							
Never	19,812 (43.7)	486 (38.4)	9,172 (39.7)	7,196 (47.8)	1,938 (49.9)	602 (51.4)	418 (47.0)
Former	12,770 (28.2)	323 (25.6)	6,280 (27.2)	4,280 (28.5)	1,221 (31.4)	372 (31.7)	294 (33.0)
Current	12,782 (28.2)	455 (36.0)	7,658 (33.1)	3,566 (23.7)	727 (18.7)	198 (16.9)	178 (20.0)
Marital status							
Never married	9,844 (21.7)	278 (22.7)	5,473 (23.7)	3,096 (20.6)	675 (17.4)	180 (15.4)	142 (16.0)
Married/partner	28,905 (63.7)	754 (59.7)	14,443 (62.5)	9,830 (65.4)	2,530 (65.1)	793 (67.7)	555 (62.4)
Separated/divorced/widowed	6,615 (14.6)	232 (18.4)	3,194 (13.8)	2,116 (14.1)	681 (17.5)	199 (17.0)	193 (21.7)
Education							
Low	15,967 (35.2)	474 (37.5)	7,600 (32.9)	5,322 (35.4)	1,587 (40.8)	520 (44.4)	464 (52.1)
Intermediate	20,323 (44.8)	562 (44.5)	10,873 (47.0)	6,547 (43.5)	1,583 (40.7)	445 (38.0)	313 (35.2)
High	9,074 (19.9)	228 (18.0)	4,637 (20.1)	3,173 (21.1)	716 (18.4)	207 (17.7)	113 (12.7)
Comorbidity							
CVD	3,318 (7.3)	102 (8.1)	1,541 (6.7)	1,114 (7.4)	356 (9.2)	108 (9.2)	97 (10.9)
CKD	1,605 (3.5)	47 (3.7)	608 (2.6)	544 (3.6)	216 (5.6)	91 (7.8)	99 (11.1)
Lung disease	6,640 (14.6)	239 (18.9)	3,476 (15.0)	2,093 (13.9)	538 (13.8)	166 (14.2)	128 (14.4)
Cancer	2,954 (6.5)	117 (9.3)	1,428 (6.2)	949 (6.3)	303 (7.8)	95 (8.1)	62 (7.0)
Diabetes	2,599 (5.7)	111 (8.8)	1,171 (5.1)	872 (5.8)	300 (7.7)	92 (7.8)	53 (6.0)
Thyroid diseaseA	3,070 (6.8)	576 (45.6)	728 (3.2)	599 (4.0)	342 (8.8)	252 (21.5)	573 (64.4)
Mean age (SD)	45,364 (100)	47.2 (14.9)	49.9 (16.7)	50.0 (15.3)	53.8 (15.8)	56.6 (15.6)	59.1 (16.4)
Mean BMI kg/m2 (SD)	45,364 (100)	26.0 (3.9)	25.9 (4.2)	26.6 (4.1)	26.9 (4.2)	27.2 (4.4)	27.4 (4.3)

^A^ In a population including individuals with thyroid disease (n=48,625).

Description of comorbid conditions; CVD - Acute myocardial infarction or stroke; CKD – Self-reported kidney disease or GFR <60 ml/min per 1.73 m^2^; Lung disease - Productive cough continuously for more than 3 months each year the last two years; Cancer – Answering ‘yes’ to having cancer in questionnaire; Diabetes - Answering ‘yes’ to having diabetes in questionnaire; Thyroid disease – Answering ‘yes’ to having thyroid disease in questionnaire.

BMI, Body Mass Index; CI, Confidence Interval; HUNT, The Trøndelag Health Study; SD, Standard Deviation; TSH, Thyroid stimulating hormone; CVD, Cardiovascular disease; CKD, Chronic kidney disease.

#### Risk of sepsis

**Figure 2:**
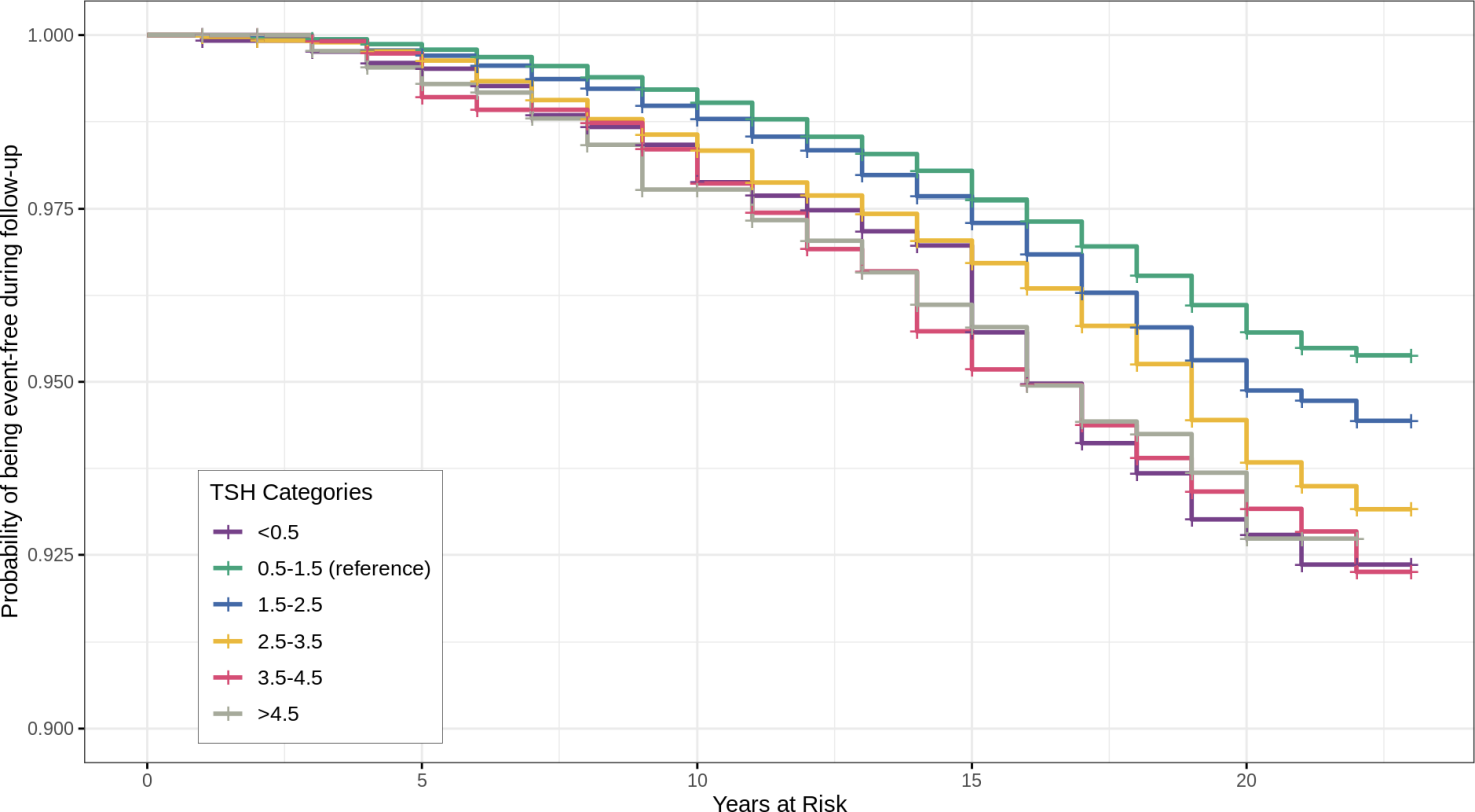
Kaplan-Meier Plot depicting probability of not experiencing an event of sepsis over time.

Participants with TSH levels <0.5 mU/L were at an increased risk of sepsis compared with the reference group (**Table 3**; **Figure 2**). There was no increased risk of sepsis for participants with TSH levels above the reference range or when analyzing TSH as continuous variable. Adjusting for comorbid conditions in sensitivity analysis did not alter the results (**Supplementary Table 2**).

**Table 3:** Cox regression analysis of the association between TSH levels and risk of sepsis.

TSH categories (mU/L)	Adjusted for age and sex^A^ HR (95% CI)	Multivariably adjusted^B^ HR (95% CI)
TSH <0.5	1.50 (1.19-1.90)	1.46 (1.16-1.85)
TSH 0.5-1.4	Reference	Reference
TSH 1.5-2.4	0.97 (0.88-1.08)	1.01 (0.92-1.12)
TSH 2.5-3.4	0.94 (0.81-1.10)	0.99 (0.85-1.16)
TSH 3.5-4.5	0.93 (0.73-1.19)	0.97 (0.76-1.25)
TSH >4.5	0.83 (0.62-1.11)	0.85 (0.63-1.14)
TSH, per unit increase	0.96 (0.90-1.02)	0.98 (0.93-1.04)

^A^ Using attained age as timescale and adjusted for sex.

^B^ Using attained age as timescale and adjusted for sex (male/female), marital status (married/partner, separated/divorced/widowed or never married), education (low/medium/high), smoking (former/current/never) and BMI (kg/m^2^).

BMI, Body Mass Index; CI, Confidence Interval; HR, Hazard ratio; TSH, Thyroid stimulating hormone.

#### Secondary analyses

The secondary analyses considered alternative exposures and outcomes. Among participants with TSH <0.5 mU/L there was an increased risk of LRTI, and a tendency of a risk of UUTI (**Supplementary Table 3**). Finally, for self-reported thyroid disease, we observed that both hyper- and hypothyroidism (or any thyroid disease) were associated with an increased risk of sepsis, but not reaching statistical significance (**Supplementary Table 4**).

### Genetic analyses

#### Risk of sepsis

**Figure 3:**
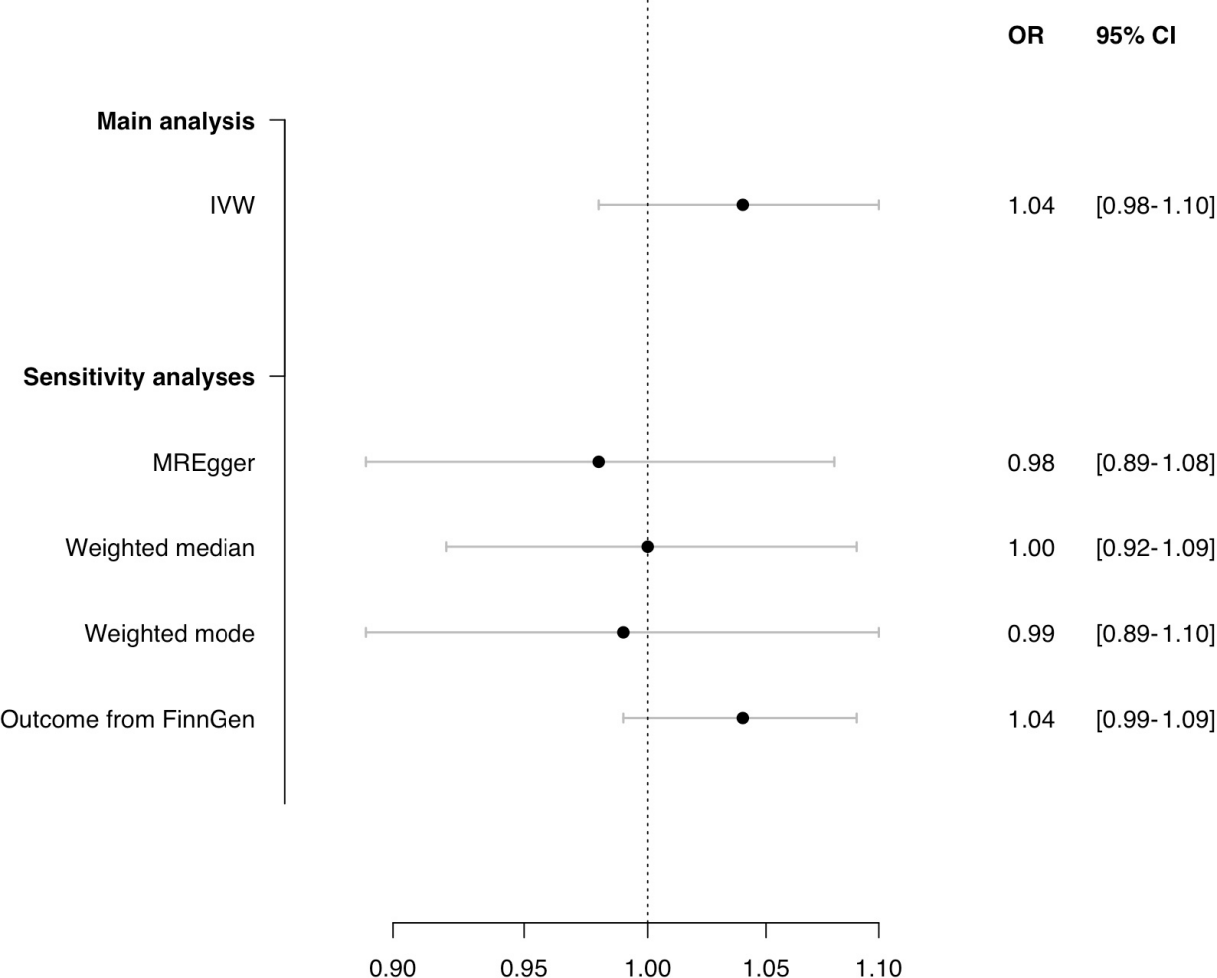
Main genetic analyses of the association between TSH levels and risk of sepsis.

Genetically predicted TSH levels within the normal range were not associated with risk of sepsis (**Figure 3**). Sensitivity analyses using MR Egger, weighted median, and weighted mode supported this null finding. Repeating the analyses using GWAS from FinnGen instead of UK Biobank yielded a similar estimate.

### Secondary genetic analyses

We investigated FT3, FT4, subgroups of FT4 (DIO and non-DIO) and TSH levels above or below reference range as alternative exposures describing thyroid function. The findings from these analyses were in line with those for TSH, showing no association between thyroid function and sepsis (**Supplementary Figure 1**). When looking at AITD as an alternative exposure, the results indicate a tendency of reduced risk of sepsis however not statistically significant. The MR Egger, weighted median and weighted mode were similar to the IVW analysis, although with wider confidence intervals included the null. In the FT4 subgroup analysis, DIO showed a tendency of higher risk compared to non-DIO, however not reaching statistical significance. For FT3, higher levels were associated with reduced risk of sepsis, but this association did not replicate using the FinnGen cohort (**Supplementary Figure 1**). We also assessed risk of LRTI and UUTI with TSH levels and found no robust evidence of association (**Supplementary Figure 2**).

## Discussion

In this study, we combined multivariable observational analyses with MR analyses and found no clear evidence that baseline TSH levels influence the risk of sepsis or common infections, LRTI and UUTI) that often precede sepsis. To our knowledge, this is the first study to triangulate prospective population-based data and MR to address whether variation in thyroid function levels above and below reference range in the general population causally affects the risk of severe infection.

Apart from our previous work on TSH and blood stream infection, we are not aware of other observational studies directly linking non-acute phase, baseline TSH levels in the general population to risk of severe infections like BSI and sepsis (10). The present analysis extends those findings by adding a genetic dimension; the lack of association in MR, together with largely null observational results within the normal TSH range, is consistent with our previous stated hypotheses that observational associations may partly reflect confounding by concurrent ill-health rather than a true causal effect. Importantly, in our previous study of TSH levels with risk of blood stream infection (10), exclusion of the first 3 and 5 years of follow-up after HUNT2 did not materially alter the association between TSH levels and BSI risk, suggesting that any confounding operates through chronic health states rather than acute illness at baseline.

We also observed no evidence of high TSH levels altering risk of sepsis in the observational analysis or in the MR analysis, contrasting the findings of an MR study by Hong et al. that reported an increased risk of certain sepsis subtypes with genetic liability to hypothyroidism (9). Several design differences may explain this discrepancy. First, our study was restricted to include subjects of only European descent, reducing population stratification bias and improving instrument validity. Secondly, Hong et al. considered diagnostic binary codes for hyper and hypothyroidism and sepsis subtypes (9), whereas we examined TSH levels above and below reference range in a general population as proxies for hypo- and hyperthyroidism. Findings by Huang et al. have interestingly suggested thyroid replacement therapy potentially protects individuals with hypothyroidism against pneumonia (8). This suggests restoring thyroid levels to the normal might reduce the risk of pneumonia. However, our findings do not indicate a risk difference in developing LRTI across varying levels of TSH. Thus, any protective effect of thyroid replacement therapy would likely need to be mediated through other mechanisms than simply normalizing TSH levels.

Our observational estimates suggested a potential increased risk of sepsis with self-reported thyroid disease, however not reaching statistical significance. When looking at the risk of sepsis in our MR analysis using genetic susceptibility to AITD as the exposure the risk was reduced, supporting our suggestion that residual confounding by concurrent ill-health might affect our results in the group that self-reported thyroid disease (10).

The most comparable observational study with population-based data investigated risk of pneumonia with hypothyroidism. Their results suggested increased risk of pneumonia with hypothyroidism but did not report on hyperthyroidism nor other thyroid measures (8). We were not able to replicate their findings in our current study, finding no robust evidence of thyroid function increasing risk of LRTI. Other studies have suggested hypothyroidism increased risk of periprosthetic joint infection (28) and urinary tract infection after female urodynamic studies (29). These studies are not directly comparable to ours as they differ greatly in design and investigated small selected clinical populations.

Previously published work has provided evidence of thyroid hormones modulating cells and mechanisms in the immune system (2, 3). As TSH is the main regulatory hormone in thyroid hormone production we hypothesized that differences in TSH would have the most prominent effect on risk of sepsis. However, we also examined FT3 and FT4, the hormones regulated by TSH, as these levels also reflect thyroid function from slightly different angles (30). We did not find robust evidence that variations in these hormones are associated with altered risk of infections in our MR analysis, and we were not able to triangulate this secondary analysis to also include observational data, as FT3 and FT4 levels were not available from a general population. Metabolism of T4 follows both DIO pathways and non-DIO pathways. DIO pathways involve enzymes activating and inactivating hormones often locally in tissue, while non-DIO pathways do not involve deiodinase enzymes but affect thyroid metabolism in other ways (31). To further see if the different pathways affected risk differently, we also stratified the SNPs associated with FT4 levels into SNP associated with DIO activity and SNPs not associated with DIO activity but found no evidence of any significant difference in risk between the two groups.

### Strength and limitations

The greatest strength of this study was the ability to triangulate the evidence across study methods, by doing this we were able to reduce the risk of residual confounding and reverse causation. The concordance of the negative results in triangulation evidence from observational and MR studies supports that there may be no causal association between thyroid function in euthyroid persons and risk of sepsis.

Another strength is the population-based design, which allowed us to examine habitual thyroid function in relation to risk of sepsis, rather than potentially suppressed TSH levels reflecting non-thyroidal illness in studies recruiting participants from clinical settings. However, in the observational analyses participants self-report of thyroid disease might have introduced recall bias, which in modern epidemiology is thought to bias the hazard ratio towards the null (32). Further, we did not have information on whether participants started treatment for potential thyroid conditions after participation. Participants with deviant thyroid hormone levels were advised to visit their general practitioners, and we also did not have repeated measurements of TSH. Lastly, in the MR analysis we are not able to formally address the possibility of non-linearity, due to limitations in the current methodology for performing such analyses (33). In theory, the observed flat linear regression line could therefore mask a J- or U-shaped association between genetically predicted TSH-levels and risk of sepsis. However, we observed no association between genetic liability to either low or high TSH-levels and risk of sepsis, which does not support a non-linear association with TSH as the exposure.

## Conclusion

Taken together, our observational and MR analyses suggest that variation in baseline thyroid function in the general adult population does not causally influence the risk of sepsis, LRTI or UUTI. In contrast to earlier MR work based on genetic liability to overt hypothyroidism in clinical populations, our findings indicate that mild deviations in thyroid function within and around the reference range are unlikely to be useful targets for sepsis prevention or risk stratification.

## Data Availability

The data analyzed in this study is subject to the following licenses/restrictions: The data underlying the observational analyses from the Trøndelag Health Study (HUNT2 and HUNT3) are governed by Norwegian data protection regulations and ethical approvals. They are available to researchers upon application to the HUNT Research Centre (https://www.ntnu.edu/hunt/research) subject to approval by the Norwegian Regional Committee for Medical and Health Research Ethics. The details on which GWAS data we used are provided in **Table 1**. Requests to access these datasets should be directed to (https://www.ntnu.edu/hunt/research) for the observational data. Different biobanks as stated in **Table 1** for GWAS data.
