# Supplementary material for: Thyroid function and risk of sepsis: a population-based prospective cohort study with traditional and genetic epidemiological analyses
Source: Front Endocrinol (Lausanne). 2026 Jun 29;17:1852413. doi: 10.3389/fendo.2026.1852413 (PMC13357225; doi:10.3389/fendo.2026.1852413)
Supplement: Supplementary file 1 [file DataSheet1.docx]

**Supplementary material**

**Thyroid function and sepsis risk: Insights from complementary epidemiological approaches**

**Supplementary Table 1**: Overview of International Classification of Diseases codes defining explicit and implicit sepsis

| **Explicit sepsis codes** | |
| --- | --- |
| A02.1, A20.7, A21.7, A22.7, A24.1, A26.7, A28.2, A32.7, A39.2, A39.4, A40, A41, A42.7, B00.7, B37.7 | |
| **Implicit Sepsis**  Any infection diagnosis in combination with at least one acute organ dysfunction codes list in the same hospital stay | |
| ***Infection codes*** | ***Acute organ dysfunction codes*** |
| A00-09, A19/28, A30-32, A36/39, A42/ 44, A46, A48/49, A54, A59, A69.0, A69.1, A69.9, A70, A74/75, A77/81, A83/89, A92/99  B00/09, B25/27, B33/34, B37/46, B48/50, B54/55, B57/58, B60, B64, B67, B95/97, B99,  G00/08, H05.0, H60.2, H70.0, J01/06, J09/22, J36, J39.0, J39.1, J85, J86, K35/37, K61, K63.0/63.1, K65, K75.0, K81.0, K83.0, L02/04, L08, M00/01, M86, M72.6, N10, N15.1, N30, N39.0, N41.0, N41.2, N41.3, N45, N70/74, N98.0, N49 O03.0, O03.5, O04.5, O08.0, O23, O75.3, O85/86, O88.3, O91, O98, U04, U07.1, U07.2 | D65, D69.5, E87.2, G93.4, I46, I95.9, J80, J95.2, J96, K72.0, K72.9, N00, N17, N99.0, R02, R09.0, R09.2, R40.0/40.2, R41, R55, R57, R57.2, R65.1 |

**Supplementary Table 2:** Cox regression analysis estimating risk of sepsis with TSH levels and adjusted for comorbid conditions.

Hazard ratios with 95% confidence intervals for risk of sepsis with TSH levels.

^A^Using attained age as timescale and adjusted for sex (male/female), marital status (married/partner, separated/divorced/widowed or never married), education (low/medium/high), smoking (former/current/never) and BMI (kg/m^2^) and comborbid conditions (cardiovascular disease - acute myocardial infarction or stroke; chronic kidney disease - self-reported kidney disease or GFR <60 ml/min per 1.73 m^2^; lung disease - productive cough continuously for more than 3 months each year the last two years; cancer – answering ‘yes’ to having cancer in questionnaire; diabetes - answering ‘yes’ to having diabetes in questionnaire; thyroid disease – answering ‘yes’ to having thyroid disease in questionnaire).

Abbreviations; BMI = Body Mass Index; CI = Confidence Interval; HR = Hazard Ratio; HUNT = The Trøndelag Health Study; TSH = Thyroid stimulating hormone

| **TSH categories (mU/L)** | **Multivariably adjusted^A^**  **HR (95%CI)** |
| --- | --- |
| TSH <0.5 | 1.43 (1.13-1.85) |
| TSH 0.5-1.4 | Reference |
| TSH 1.5-2.4 | 1.02 (0.92-1.12) |
| TSH 2.5-3.4 | 0.99 (0.85-1.16) |
| TSH 3.5-4.5 | 0.98 (0.76-1.25) |
| TSH >4.5 | 0.85 (0.63-1.14) |
| TSH, per unit increase | 0.98 (0.93-1.04) |

**Supplementary Table 3:** Cox regression analyses of the association between TSH levels and risk of LRTI and UUTI

| **TSH categories (mU/L)** | **LRTI**  **HR (95%CI)** | **UUTI**  **HR (95%CI)** |
| --- | --- | --- |
| TSH <0.5 | 1.20 (1.02-1.41) | 1.10 (0.85-1.42) |
| TSH 0.5-1.4 | Reference | Reference |
| TSH 1.5-2.4 | 0.97 (0.91-1.03) | 0.92 (0.83-1.02) |
| TSH 2.5-3.4 | 0.95 (0.86-1.06) | 0.83 (0.71-0.97) |
| TSH 3.5-4.5 | 0.96 (0.82-1.13) | 0.71 (0.54-0.92) |
| TSH >4.5 | 0.96 (0.81-1.15) | 0.76 (0.58-1.02) |
| TSH, per unit increase | 1.00 (0.98-1.02) | 0.97 (0.95-1.01) |

Hazard ratios with 95% confidence intervals for risk of LRTI and UUTI with TSH levels categorized. Using attained age as timescale and adjusted for sex (male/female), marital status (married/partner, separated/divorced/widower or never married), education (low/medium/high), smoking (former/current/never) and BMI (kg/m^2^).

Abbreviations: BMI=Body Mass Index; CI=Confidence intervals; HR=Hazard ratio; LRTI=Lower respiratory tract infections; TSH=Thyroid stimulating hormone; UUTI=Upper urinary tract infection

**Supplementary Table 4:** Cox regression analysis estimating risk of sepsis with self-reported thyroid disease

Hazard ratios with 95% confidence intervals for risk of sepsis with self-reported thyroid disease in HUNT. Using attained age as timescale and adjusted for sex (male/female), marital status (married/partned, separated/divorced/widowed or never married), education (low/medium/high), smoking (former/current/never) and BMI (kg/m^2^).

Abbreviations; BMI = Body Mass Index; CI = Confidence Interval; HR = Hazad Ratio; HUNT = The Trøndelag Health Study; TSH = Thyroid stimulating hormone

| **Exposure** | **HR (95% CI)** |
| --- | --- |
| Self-reported thyroid disease | 1.10 (0.93-1.29) |
| Self-reported hyperthyroidism | 1.22 (0.97-1.53) |
| Self-reported hypothyroidism | 1.10 (0.94-1.30) |

Results from secondary Mendelian Randomization analyses describing odds ratios and 95% confidence intervals for risk of sepsis, with different exposures describing thyroid function. Each exposure was analyzed using the IVW, MR Egger, weighted median and weighted mode method.

Abbreviations: AITD=Autoimmune thyroid disease; CIs=Confidence intervals; DIO=Deiodenase; IVW=Inverse Variance Weighted; MR= Mendelian randomization; OR=Odds ratio; TSH=Thyroid stimulating hormone;

**Supplementary Figure 1:** Genetic analysis investigating risk of sepsis with alternative exposures associated with thyroid function.
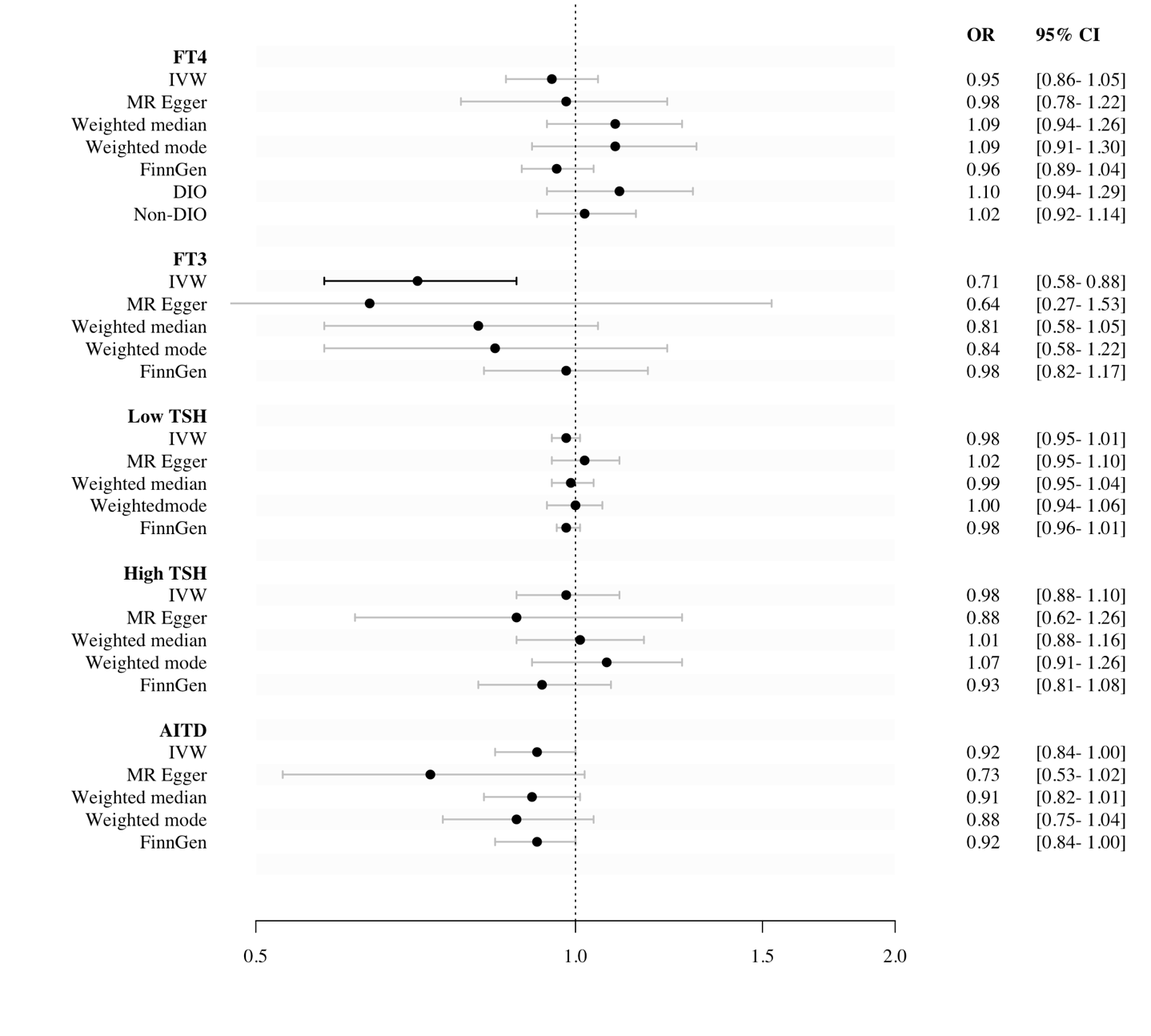


**Supplementary Figure 2:** Genetic analysis investigating risk of LRTI and UUTI with TSH levels.


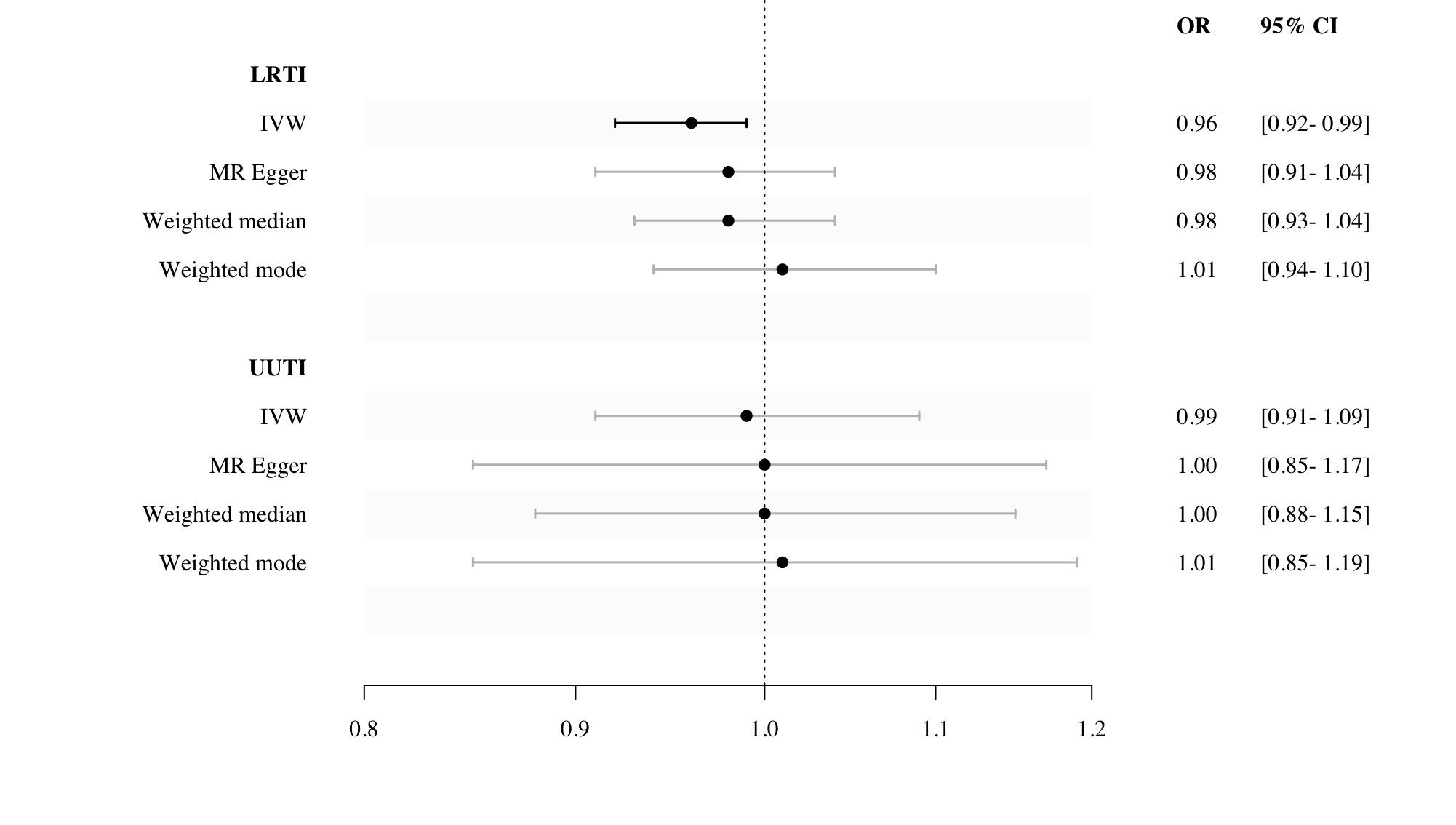


Results from secondary Mendelian Randomization analyses describing odds ratios and 95% confidence intervals for risk of Lower Respiratory Tract Infection (LRTI), Upper Urinary Tract Infection, with Thyroid stimulating Hormon levels Each exposure was analyzed using the IVW, MR Egger, weighted median and weighted mode method. Odds ratios and 95% confidence intervals for risk of LRTI and UUTI with TSH levels within the normal range.

Abbreviations: AITD=Autoimmune thyroid disease; CIs=Confidence intervals; IVW=Inverse Variance Weighted; LRTI=Lower urinary tract infections; MR= Mendelian randomization; OR=Odds ratio; TSH=Thyroid stimulating hormone; UUTI=Upper urinary tract infection.
